# Associations of Sleep, Screen Time, and Extracurricular Activities With Cognitive Development: A Longitudinal Study

**DOI:** 10.1002/jad.70069

**Published:** 2025-11-04

**Authors:** Jiayi Zheng, Emma Berg, Michelle L. Byrne, Divyangana Rakesh

**Affiliations:** ^1^ Neuroimaging Department, Institute of Psychology, Psychiatry & Neuroscience King's College London London UK; ^2^ Melbourne Graduate School of Education The University of Melbourne Victoria Melbourne Australia; ^3^ School of Psychological Sciences Monash University Clayton Victoria Australia

**Keywords:** behavioral science, child development, cognitive development, developmental psychology, extracurricular activities, public health, screen time, sleep

## Abstract

**Introduction:**

Adolescence is a sensitive period typified by marked cognitive and neural development, during which modifiable lifestyle factors may be particularly relevant. However, longitudinal associations of modifiable lifestyle factors—including sleep, screen time, and extracurricular activities—with cognitive development over time remain to be investigated, leaving the directionality of these relationships unclear.

**Methods:**

We used baseline and 2‐year follow‐up data (*n* = 7043) from the Adolescent Brain Cognitive Development (ABCD) study. Linear mixed‐effect models were employed to assess the association of modifiable lifestyle factors with the development of different cognitive functions over time. We additionally examined the moderating role of sex in these associations.

**Result:**

Longer sleep duration, greater time spent on nonphysical activities, and shorter duration of physical activity and screen usage—across passive watching, social media, and social engagement—were significantly associated with greater increases in cognitive scores. Sex moderated the association between passive screen watching and the duration of physical extracurricular activities with inhibitory control and attention. The negative association between passive watching and inhibitory control and attention development was stronger in females.

**Conclusion:**

Our findings suggest that modifiable lifestyle factors are associated with adolescent cognitive development and point to the potential of lifestyle‐based interventions to support optimal development during this formative period.

## 1 Introduction

Cognitive development during childhood and adolescence is a crucial predictor for children's educational and social outcomes, including academic performance (Peng and Kievit 2020). Consequently, researchers have shown an interest in studying the development of cognitive functioning over the past decade, with a focus on the mechanisms, factors, and interventions for improving cognitive functioning (Buschkuehl and Jaeggi 2010). In particular, adolescence, a period typified by dynamic neurobiological development (Rakesh et al. 2024), is an important period for the development of cognitive abilities (Larsen and Luna 2018). Importantly, research has shown that lifestyle factors such as physical activity, sleep health, and screen time may relate to cognitive abilities during this period (Herting and Chu 2017; Jirout et al. 2019). However, there is a preponderance of cross‐sectional studies, and little is known about how these lifestyle factors may be associated with development of cognitive abilities *over time*. Given that adolescence is characterized by substantial biological, social, and behavioral changes, and heightened neural plasticity (Dahl et al. 2018), it is essential to investigate how lifestyle factors may associate with cognitive skills during this key developmental period. Such insights could inform targeted interventions aimed at optimizing cognitive performance and helping adolescents reach their full potential.

Adolescence is marked by significant changes in cognitive function, including improvements in fluid cognitive functioning (FCF), which is the capacity to reason and solve novel problems and includes specific constructs such as executive function, processing speed, problem solving, and memory (Crone et al. 2017; Frischkorn et al. 2014; Kail 1991; Luciana et al. 2018). Adolescence is also characterized by increases in crystallized cognitive functioning (CCF), which involves the use of acquired knowledge, skills, and experience and includes domains such as language ability, reading comprehension, and literacy and numeracy skills (Eckert 2004; Geary 2000; Kolić‐Vehovec et al. 2014; Sullivan and Brown 2015). This period is also characterized by a renewed period of brain plasticity, making the brain particularly sensitive to both biological and environmental factors that are related to long‐lasting patterns in cognition, emotion, and social behavior (Larsen and Luna 2018; Rakesh et al. 2024). Importantly, while cognitive function is moderately heritable (Devlin et al. 1997), various environmental and lifestyle factors, such as sleep, screen time, and participation in extracurricular activities are thought to play a key role in shaping cognitive development during this time (Herting and Chu 2017). Understanding how these factors are associated with cognitive development in adolescence is crucial.

Sleep has consistently been reported as being associated with adolescents' cognitive function. Sufficient sleep contributes to higher general cognitive ability and better academic performance (Rey et al. 2020). Conversely, poor sleep quality and shorter sleep duration can lead to impairments in inhibitory control and attention (Vriend et al. 2012) and working memory (Steenari et al. 2003). In addition, excessive screen time is associated with lower cognitive performance (Liu et al. 2022). However, different types of screen use (e.g., social media vs. gaming) may have varied relationships. Indeed, some types of screen use, such as passive TV viewing and social networking (including social media use, texting, and video calling), are associated with lower language ability, verbal intellect, inhibitory control and attention, and learning (Johnson et al. 2007; Loh and Kanai 2016; Wegmann et al. 2020). In contrast, gaming has been shown to associate with improving visual memory, inhibitory control and attention, visuospatial memory, and reading ability (Bediou et al. 2018; Özçetin et al. 2019; Rakimahwati and Roza 2020). Finally, participation in extracurricular activities has been found to be associated with positive cognitive outcomes, such as increased inhibitory control and attention, self‐regulation, and problem‐solving abilities (Farb and Matjasko 2012). For example, engaging in physical activities is linked to higher cognitive scores (Esteban‐Cornejo et al. 2015), while lack of engagement in extracurricular activities has been associated with lower executive function (Bidzan‐Bluma and Lipowska 2018). Additionally, participation in nonphysical activities such as music, drama, and art have also shown associations with higher cognitive performance and academic achievement (Jamey et al. 2024; Winsler et al. 2020; Zuk et al. 2014). Overall, these findings underscore the significant role sleep, screen time, and extracurricular activities play in cognitive development.

Providing further support for the role of these factors, a recent cross‐sectional study using a large population‐based dataset from the Adolescent Brain Cognitive Development (ABCD) Study, the same sample as that leveraged in the present study, found that sleep, extracurricular activities, and screen time were associated with adolescents’ fluid cognition (Kirlic et al. 2021). However, while previous research has found associations between various modifiable lifestyle factors and cognitive abilities, most studies have used small and/or cross‐sectional samples, which limit the generalizability of the results and preclude inferences about directionality. That is, it remains unknown whether these modifiable lifestyle factors *precede* the assessed cognitive outcomes (Kirlic et al. 2021). For example, it is possible that instead of screen time contributing to lower cognitive performance, individuals with lower cognitive performance engage more with screens. While some evidence suggests that specific physical training programs positively associate with cognitive development (Sallis et al. 1999), previous work has limited external validity. They primarily focus on structured interventions rather than assessing the general physical activities adolescents typically engage in, such as recreational sports, dance, or ballet. Moreover, many of these studies tend to focus on either overall cognitive function (e.g., Liu et al. 2022; Rey et al. 2020) or few specific cognitive domains (e.g., Bediou et al. 2018; Rakimahwati and Roza 2020), overlooking the possibility that different modifiable lifestyle factors may have unique associations with specific cognitive domains, such as attention, memory, or executive function. Further research using longitudinal designs to clarify the directionality of these relationships is needed.

Finally, previous literature has demonstrated differences in cognitive development between males and females (Andreano and Cahill 2009). During adolescence, girls tend to score higher on cognitive tests than boys, but their cognitive development decelerates relative to boys from the age of 14–15 onwards (Colom and Lynn 2004). Further, males perform better in spatial tasks (Levine et al. 2016), while females excel in verbal intellect (Scheuringer and Pletzer 2017) and declarative memory tasks (Maitland et al. 2004). Additionally, there are also sex differences in sleep patterns, screen time usage, and extracurricular activities during adolescence. For example, boys engage in more physical activities (McKenzie et al. 2000), less nonphysical activities (Green 1993), spend more time watching TV (Ozmert et al. 2002) and gaming (Kaliebe and Weigle 2018), and wake up later than girls (Loessl et al. 2008; Merdad et al. 2014). Given these differences, it is possible that sex moderates the association between modifiable lifestyle factors and cognitive development. However, longitudinal research on this topic is lacking.

To address these gaps in the literature, the present study aims to explore the association of modifiable lifestyle factors (i.e., sleep, extracurricular activities, and screen time) with cognitive development during adolescence using a large longitudinal sample from the ABCD study. Additionally, in exploratory analyses, we investigated sex differences in these associations. Based on previous work (e.g., Bediou et al. 2018; Esteban‐Cornejo et al. 2015; Rey et al. 2020; Walsh et al. 2020; Winsler et al. 2020), we hypothesized that higher duration of passive watching, social media use, and social engagement would be associated with lower increases in cognitive scores over time. On the other hand, we expected that higher duration of gaming would be associated with greater increases in cognitive scores across domains. Additionally, we hypothesized that better sleep quality (characterized by longer sleep duration and less difficulty falling asleep) and greater involvement in physical and nonphysical extracurricular activities would be associated with greater increases in cognitive scores over time. While we expected sex differences in these associations, given the lack of prior work in this area, we did not make specific hypotheses for our exploratory aim.

## 2 Methods and Materials

### 2.1 Participants

Participants were from the ongoing ABCD study (https://abcdstudy.org/; baseline assessment of ABCD [release 5.1]). This extensive longitudinal study has recruited over 11,500 children aged 9–11 years across 21 sites in the United States, examining their psychological and neurobiological development from early adolescence to early adulthood. The present study utilized data from both the baseline and 2‐year follow‐up. To ensure the recruitment efforts maximized representation of U.S. demographic and socioeconomic characteristics, the sampling design oversampled specific groups to address under‐representation (Garavan et al. 2018). Ethics approval for the ABCD Study was obtained from the central Institutional Review Board at the University of California, San Diego, as well as from local IRBs (Auchter et al. 2018). Only participants who had complete data on all variables of interest (i.e., cognitive scores at both time points, modifiable lifestyle factors, and covariates) were included in analyses, resulting in a final sample of 7043 adolescents.

### 2.2 Measures of Modifiable Lifestyle Factors

Modifiable lifestyle factors included sleep, screen time, and physical and nonphysical extracurricular activities. Histograms and correlation plots are available in the Supplement.

#### 2.2.1 Sleep

Sleep was measured using the parent‐reported Sleep Disturbance Scale for Children (Bruni et al. 1996; Ferreira et al. 2009). Two key factors were evaluated: sleep duration (“how many hours of sleep the child has per night;” values in hours) and sleep initiation difficulties (“the amount of time taken to fall asleep;” values in minutes). Higher values for sleep duration indicate that children sleep for longer periods, while higher values for sleep initiation suggest taking longer to fall asleep.

#### 2.2.2 Screen Time

Screen time was measured using the self‐report ABCD Study Screen Time Questionnaire (Andreassen et al. 2012). Participants reported their screen time for various media types, with response options ranging from “none” to “more than 4 h.” A weighted average daily use variable was created based on average weekday and weekend screen time. We categorized these different media uses into four distinct types: Passive watching (“how many hours do you: Watch TV shows or movies?”, “how many hours do you: Watch videos (such as YouTube)”); Gaming (“how many hours do you: Play video games on a computer, console, phone or other device [Xbox, Play Station, iPad]”); Social engagement (“how many hours do you: Text on a cell phone, tablet, or computer [e.g. GChat, Whatsapp, etc.]”, “how many hours do you: Video chat [Skype, Facetime, etc.]”); and Social media (“how many hours do you: Visit social networking sites like Facebook, Twitter, Instagram, etc.”).

#### 2.2.3 Extracurricular Activities

Children's participation in extracurricular activities was assessed using the parent‐report ABCD Study Sports and Activities Involvement Questionnaire (Huppertz et al. 2016). This questionnaire assessed children's lifetime history of activity involvement, the frequency and duration of their participation, and their activity level over the past year. We calculated the total hours per week spent engaging in (1) physical activities and (2) nonphysical activities during the previous twelve months for each participant. See the Supporting Information for activities included in the physical and nonphysical categories and the calculation method.

### 2.3 Measures of Cognitive Functioning

Cognitive function was measured using the NIH Cognition Battery (Luciana et al. 2018; McDonald 2014). This battery comprises tests that assess six cognitive abilities: attention, executive function, working memory, episodic memory, language, and processing speed (Akshoomoff et al. 2013). It has been shown to have strong reliability and validity in adolescent samples (Mungas et al. 2013; Weintraub et al. 2013). Given that the focus of this study is on longitudinal changes over time, a subset of five tasks that were administered at both baseline and the 2‐year follow‐up were used.

Although analyzed separately, these cognitive abilities fall into two broad constructs: FCF and CCF. FCF reflects reasoning, processing speed, and problem‐solving abilities independent of acquired knowledge (Kent 2017). FCF was assessed using three tasks from the NIH Toolbox: (1) Inhibitory Control and Attention (measured using Flanker task), (2) Visuospatial Sequencing and Memory (measured using Picture Sequence Memory task), (3) Information Processing Speed (measured using Pattern Comparison Processing Speed task). :CCF refers to acquired knowledge and verbal abilities shaped by past learning and cultural experience (Cattell 1987). This was assessed using two tasks: (1) Verbal Intellect (measured using Picture Vocabulary task), (2) Reading and Language Ability (measured using Oral Reading Recognition task). The List Sorting Working Memory and Dimensional Change Card Sort tasks were not administered at the 2‐year follow‐up and thus could not be analyzed in the present study.

The broader constructs of fluid and crystallized cognitive functioning (FCF and CCF, respectively) were used solely for interpretation purposes, as FCF is generally more susceptible to environmental influences than CCF (Blair 2006). This conceptual distinction provided a framework for understanding differential associations between modifiable lifestyle factors and specific cognitive domains. Moreover, individual task scores were used as only a subset of tasks were administered at follow‐up, which precluded the calculation of composite scores. Age‐uncorrected scores were used for analyses given our focus on development. See Table 1 for the detailed mapping of tasks to cognitive domains and FCF/CCF classification. See Supporting Information for more detail (Figure S1 for histogram and Figure S2 for correlation plot).

**TABLE 1:** The NIH Toolbox cognitive battery.

Task	Cognitive functions	Cognitive functioning categorization
NIH Toolbox Flanker®	Inhibitory Control and Attention	FCF
NIH Toolbox Pattern Comparison Processing Speed®	Information Processing Speed	
NIH Toolbox Picture Sequence Memory Test®	Visuospatial sequencing and memory	
NIH Toolbox Picture Vocabulary Test®	Verbal intellect	CCF
NIH Toolbox Oral Reading Recognition Test®	Reading and Language Ability	

### 2.4 Statistical Methods

Residual change score models were used to assess the association of modifiable lifestyle factors (i.e., passive watching, gaming, social media, social engagement, physical and nonphysical extracurricular activities, sleep duration, and sleep initiation) and changes in cognitive scores over time using linear mixed effects models (*lmer* package in R version 2023.09.0.). All modifiable lifestyle and cognitive variables were analyzed as individual constructs rather than as composite or multi‐item scales. Individual NIH Toolbox task scores were used in all statistical analyses, rather than composite scores. Residual change score models were leveraged as they are less prone to measurement error when data from only two time points are available (Bergh and Fairbank 2002). As we did not impute missing data, only complete cases were included in each analysis. We modeled cognitive function scores at T2 as the dependent variable, with each modifiable lifestyle factor as the predictor in separate models (resulting in eight models for each outcome variable). To capture changes in cognitive function, we included the corresponding cognitive function score at T1 as an additional predictor in the model. We covaried for sex, age at T2, age difference between T2 and T1, income‐to‐needs ratio (calculated as income relative to the federal poverty threshold for the respective household size) and average parent educational attainment (in years) in the models, as socioeconomic status is known to be associated with cognitive function (Lawson et al. 2018; Rakesh et al. 2025a). We additionally accounted for visit type at T2 (remote vs. in‐person vs. hybrid). Family ID was modeled as a random effect to account for multiple children from the same family. We applied False Discovery Rate (FDR) correction (pFDR < 0.05) separately for each outcome variable, correcting across tests run for each of the eight predictors (i.e., modifiable lifestyle factors). Further, in exploratory analyses, we tested whether sex moderated these associations.

## 3 Results

See Table 2 for descriptive information.

**TABLE 2:** Descriptive information

		*n* or Mean (SD)	
		Baseline	2‐year follow‐up
No. of participants		7043*, 52.2% male, 47.8% female	
Age (months)		118.98 (7.50)	144.32 (8.03)
Income‐to‐needs ratio		3.46 (2.28)	
Average parent education years		15.08 (2.63)	
Modifiable lifestyle factors (hours)	Passive watching	0.97 (1.72)	
	Gaming	1.01 (1.07)	
	Social media	0.10 (0.38)	
	Social engagement	0.37 (0.79)	
	Physical extracurricular activities	7.19 (7.18)	
	Nonphysical extracurricular activities	1.99 (3.66)	
	Sleep duration	9.08 (1.05)	
	Sleep initiation	0.40 (0.19)	
Cognition task ‐ FCF (scores)	NIH Toolbox Flanker	94.55 (8.71)	100.36 (7.49)
	NIH Toolbox Picture Sequence Memory Test	103.52 (12.09)	109.55 (12.14)
	NIH Toolbox Pattern Comparison Processing Speed	88.52 (14.37)	103.75 (15.09)
Cognition task ‐ CCF (scores)	NIH Toolbox Picture Vocabulary Test	85.25 (7.99)	89.42 (8.31)
	NIH Toolbox Oral Reading Recognition Test	91.32 (6.73)	95.03 (6.50)

*Note:* Raw scores have been provided for the NIH cognitive battery. *100% in person in baseline year, 92.3% in person, 0.3% remotely, 7.4% hybrid at the 2‐year follow‐up.

Descriptive Statistics of Participant Characteristics and Study Variables at Baseline and Follow‐Up.

### 3.1 Normative Change in Cognitive Function

Across the whole sample, we found that cognitive scores across all five tasks increased significantly with age (see Supporting Information: Table S1 for model output). As such, positive coefficients from residual change score models (i.e., models testing associations between modifiable lifestyle factors and cognitive development) represent greater increases as a function of the modifiable lifestyle factor, and negative coefficients indicate lower increases.

### 3.2 Association Between Modifiable Lifestyle Factors and Cognitive Development

In our main analysis, we found that all modifiable lifestyle factors, with the exception of sleep initiation difficulties, were associated with changes in at least one domain of cognitive function. In general, more screen time (across types) and greater duration of physical activities were associated with lower increases in cognitive scores. On the other hand, greater duration of nonphysical extracurricular activities and longer sleep duration were associated with greater increases in cognitive scores. Specific associations are described in greater detail below.

#### 3.2.1 FCF Development

Greater passive watching and social media use were associated with lower increases for all three outcome variables of FCF. Higher sleep duration was associated with greater increases for all three outcomes of FCF. Moreover, more time spent on social engagement was associated with lower increases in inhibitory control and attention and visuospatial sequencing and memory. Further, greater duration of nonphysical extracurricular activities was associated with greater increases in inhibitory control and attention. See Table 3 for model output and Supporting Information (Table S2) for additional metrics including semi‐partial R², conditional R², AIC, and BIC.

**TABLE 3:** Model output for associations between modifiable lifestyle factors and FCF development.

Predictor	Outcome	B	SE	*t* value	*p*	pFDR
Passive watching	Inhibitory control and attention	−0.22	0.05	−4.72	< 0.001	0.010*
Gaming		−0.08	0.08	−0.98	0.329	0.376
Social media		−0.50	0.21	−2.40	0.017	0.026*
Social engagement		−0.50	0.10	−4.96	< 0.001	0.006*
Physical		−0.01	0.01	−1.11	0.265	0.354
Nonphysical		0.06	0.02	2.63	0.008	0.017*
Sleep duration		0.33	0.08	4.09	< 0.001	0.119*
Sleep initiation		0.00	0.01	−0.68	0.498	0.498
Passive watching	Information processing speed	−0.40	0.09	−4.39	< 0.001	< 0.001*
Gaming		−0.15	0.15	−0.98	0.325	0.371
Social media		−0.99	0.41	−2.44	0.015	0.039*
Social engagement		−0.35	0.20	−1.80	0.072	0.144
Physical		−0.01	0.02	−0.52	0.604	0.604
Nonphysical		0.05	0.04	1.11	0.266	0.355
Sleep duration		0.46	0.15	2.97	0.003	0.012*
Sleep initiation		−0.02	0.01	−1.70	0.090	0.144
Passive watching	Visuospatial sequencing and memory	−0.25	0.07	−3.52	< 0.001	0.001*
Gaming		−0.07	0.11	−0.62	0.538	0.696
Social media		−1.40	0.30	−4.68	< 0.001	< 0.001*
Social engagement		−0.90	0.14	−6.32	< 0.001	< 0.001*
Physical		−0.01	0.02	−0.51	0.609	0.696
Nonphysical		0.03	0.03	0.92	0.358	0.572
Sleep duration		0.45	0.12	3.85	< 0.001	< 0.001*
Sleep initiation		−0.00	0.01	−0.28	0.776	0.776

*Note:* * = *p* < 0.05. For effect sizes and model fit indices (semi‐partial R², conditional R², AIC, BIC), see Supporting Information (Table S2).

#### 3.2.2 CCF Development

There were more modifiable lifestyle factors associated with the development of CCF compared to FCF. Generally, longer duration of passive watching, social media use, and social engagement, as well as physical activity, were associated with lower increases in reading and language ability and verbal intellect. In contrast, greater time spent on nonphysical activities and longer sleep duration were associated with higher increases in both cognitive domains. Further, more time spent on gaming was associated with lower increases in verbal intellect, and higher increases in reading and language ability. See Table 4 for model output and Supporting Information (Table S3) for additional metrics including semi‐partial R², conditional R², AIC, and BIC.

**TABLE 4:** Model output for associations between modifiable lifestyle factors and CCF development.

Predictor	Outcome	B	SE	*t* value	*p*	pFDR
Passive watching	Verbal intellect	−0.17	0.04	−4.87	< 0.001	< 0.001*
Gaming		−0.12	0.06	−2.06	0.040	0.045*
Social media		−0.79	0.15	−5.26	< 0.001	< 0.001*
Social engagement		−0.57	0.07	−8.03	< 0.001	< 0.001*
Physical		−0.05	0.01	−5.66	< 0.001	< 0.001*
Nonphysical		0.07	0.02	4.11	< 0.001	< 0.001*
Sleep duration		0.33	0.06	5.63	< 0.001	< 0.001*
Sleep initiation		0.00	0.01	0.31	0.754	0.754
Passive watching	Reading and language ability	−0.08	0.03	−3.04	0.002	0.004*
Gaming		0.09	0.04	2.10	0.036	0.041*
Social media		−0.28	0.11	−2.46	0.014	0.019*
Social engagement		−0.19	0.05	−3.39	0.001	0.001*
Physical		−0.03	0.01	−4.33	< 0.001	< 0.001*
Nonphysical		0.06	0.01	5.06	< 0.001	< 0.001*
Sleep duration		0.18	0.05	3.95	< 0.001	< 0.001*
Sleep initiation		−0.00	0.00	−1.00	0.316	0.316

*Note:* * = *p* < 0.05. For effect sizes and model fit indices (semi‐partial R², conditional R², AIC, BIC), see Supporting Information (Table S3).

### 3.3 Sex Differences in the Association Between Modifiable Lifestyle Factors and Cognitive Development

**FIGURE 1:**
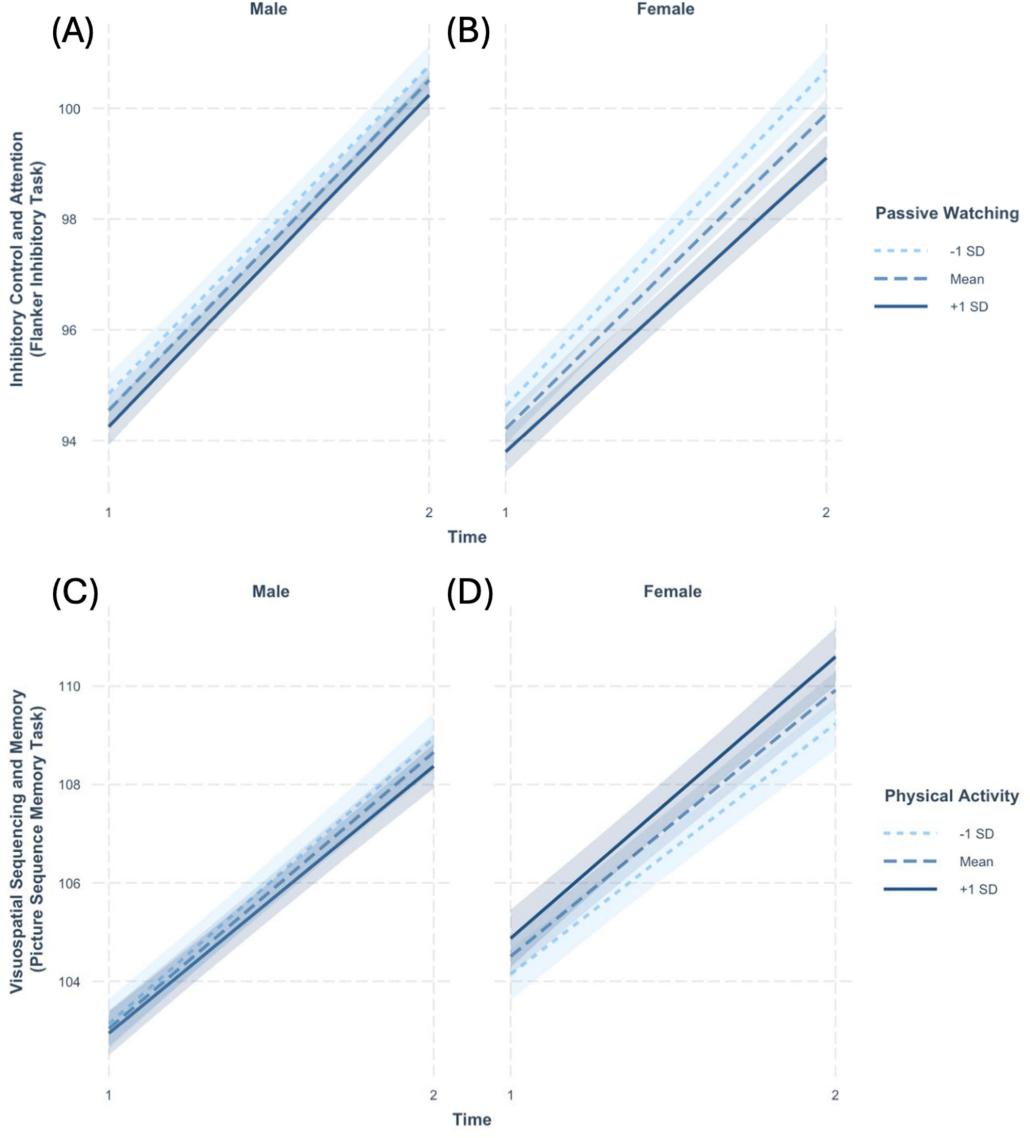
The role of sex as a moderator in the association between modifiable lifestyle factors and cognitive development. Interaction between passive screen use and time predicting inhibitory control and attention (performance on the Flanker Task) for males (A) and females (B). Interaction between physical activity and time predicting visuospatial sequencing and memory (performance on the Picture Sequence Memory Task) for males (C) and females (D). Lines represent estimated performance at −1 SD, mean, and +1 SD levels of the respective moderator (passive watching in A–B; physical activity in C–D). Shaded regions indicate 95% confidence intervals.

We found that sex moderated the associations of passive watching and inhibitory control and attention (B = −0.28, SE = −3.03, pFDR = 0.002). Specifically, females exhibited lower increases as a function of greater passive watching (B = −0.38, SE = 0.07, *p* ≤ 0.001) than males (B = −0.17, SE = 0.07, *p* = 0.010). See Table 5 for model output and Supporting Information (Table S6) for additional metrics including semi‐partial R², conditional R², AIC, and BIC. See Figure 1A,B for the interaction plot.

**TABLE 5:** Model output for the associations by sex.

Predictor	Outcome	Sex	B	SE	*t* value	*p*
Passive watching	Inhibitory control and attention	Male	−0.17	0.07	−2.65	0.008*
		Female	−0.38	0.07	−5.60	< 0.001*
Physical extracurricular activities	Visuospatial sequencing and memory	Male	−0.03	0.02	−1.50	0.135
		Female	0.05	0.03	1.90	0.058

*Note:* * = *p* < 0.05. For effect sizes and model fit indices (semi‐partial R², conditional R², AIC, BIC), see Supporting Information (Table S6).

We also found that sex moderated the association of physical extracurricular activities and visuospatial sequencing and memory (B = 0.10, SE = 0.03, pFDR = 0.022). However, results for sex stratified analyses were not statistically significant within the female (B = 0.05, SE = 0.03, *p* = 0.058) or male (B = −0.03, SE = 0.02, *p* = 0.135) sample. Sex did not significantly moderate any other associations. See Table 5 for model output and Supporting Information (Table S6) for additional metrics including semi‐partial R², conditional R², AIC, and BIC. See Figure 1C,D for the interaction plot. See Supporting Information (Tables S4, 5) for the model output for nonsignificant results.

## 4 Discussion

This study aimed to explore the association of modifiable lifestyle factors—including sleep, extracurricular activities, and screen time—with cognitive development during adolescence using longitudinal data. Additionally, we aimed to investigate whether sex moderated these associations. We found that, in general, longer sleep duration, greater duration of nonphysical activities, shorter duration of physical activities, and less time spent on passive watching, social media, and social engagement were associated with greater increases in cognitive functioning. Furthermore, duration of physical activity and passive watching were associated with cognitive development differently in males and females.

In line with our hypotheses, longer sleep duration was linked to greater increases in cognitive functioning across all tasks. This is consistent with cross‐sectional literature linking longer sleep duration with higher executive function and information processing speed (Lo et al. 2019; Rossa et al. 2014; Taveras et al. 2017), and academic and verbal abilities (Agostini and Centofanti 2024; Perkinson‐Gloor et al. 2013). Our findings extend prior cross‐sectional work by demonstrating the benefits of longer duration over time during early adolescence. These cognitive benefits may be due to the role of slow‐wave sleep in memory consolidation processes (de Bruin et al. 2017), which supports long‐term memory formation (Rasch and Born 2013), language learning (Rasch 2017), and information processing (Cowan et al. 2021). Our findings offer critical empirical support for policies such as delayed school start times, which better align with natural sleep patterns and reduce absences, and have been shown to improve academic outcomes (Edwards 2012; Wahlstrom et al. 1954).

In contrast to prior cross‐sectional work (Kirlic et al. 2021), we found no evidence that sleep initiation is related with cognitive development. Instead, our findings suggest that sleep duration may be more critical than initiation difficulties for cognitive changes in early adolescence. Given that sleep patterns and circadian rhythms change during adolescence (Dahl and Lewin 2002), variations in sleep habits may mask the association between initiation difficulties and cognition.

Longer time spent on passive watching, social media, and social engagement was found to be associated with lower cognitive improvements, in line with previous literature on the adverse relationship between excessive screen use and executive functioning, attention regulation, and academic performance in adolescents (Marciano et al. 2021; Walsh et al. 2020). However, associations differed based on the type of screen usage and the cognitive task. Specifically, we found that more passive watching was associated with lower increases in scores across all five cognitive domains. This aligns with previous literature suggesting that more passive screen time, such as watching TV, negatively associates with inhibitory control and attention, reading and language ability, working memory, and academic skills (Christakis et al. 2004; Hanson 2017; Johnson et al. 2007). This may be explained by the “overstimulation hypothesis,” which suggests that prolonged intense audiovisual stimulation may overwhelm the brain, impairing plasticity and reducing attention capacity (de Sousa Lima et al. 2021; Ravinder et al. 2016; Staats et al. 2024). However, content type matters—educational programming, for instance, can promote cognitive development, particularly in literacy and numeracy (Mares and Pan 2013). Future work should focus on distinguishing between different types of content, investigating how genres, such as educational versus entertainment, are related with cognitive function.

Similarly, greater social media use was linked to lower cognitive improvements across domains. Our finding could be explained by the cognitive costs of media multitasking, which has been linked to impairments in executive function and learning (Baumgartner et al. 2014; Cain et al. 2016; Junco 2012). This, in turn, could hinder emotion regulation (Ahmed et al. 2015; Zelazo and Cunningham 2007), which may partly explain the association between increased social media use and higher mental health problems (Giordano et al. 2023; Keles et al. 2020; Twenge et al. 2022). Our findings highlight the importance of policy to effectively manage social media use and support adolescents' cognitive development, which should be developed through collaboration among governments, parents, and educators (Goodyear et al. 2025; O'Keeffe et al. 2011).

Consistent with our hypotheses and prior research (Aharony and Zion 2019; Lister‐Landman et al. 2017; Loh and Kanai 2016), longer duration of screen‐based social engagement was also associated with lower improvements in cognitive abilities, except for information processing speed. We speculate that this may be due to the need for rapid shifts in attention, which may deplete cognitive resources (Loh and Kanai 2016). However, some evidence suggests that online social engagement can improve language and literacy skills (Li et al. 2015), but differences in the population studied (US adolescents vs. second language English learners) and the operational definition of social engagement used may explain these inconsistencies. Thus, the association between social engagement and cognitive abilities may depend on activity type and content.

Gaming, on the other hand, was positively associated with the development of reading and language ability but negatively associated with the development of verbal intellect. These findings align with evidence that gaming is associated with enhanced concentration and reading efficiency (Mohd Rosli and Fadhlullah 2023; Ostiz‐Blanco et al. 2021; Pasqualotto et al. 2022) but stand in contrast to prior work that shows gaming to be associated with enhanced verbal intellect (DeHaan et al. 2010; Vahdat and Behbahani 2013). One possible reason would be that the participants in those studies were young adults and language‐learners, playing games related specifically with vocabulary acquisition (e.g., language music video games and a game called “Run Away”). Moreover, in contrast to prior work (Bediou et al. 2018; Dale et al. 2020), we found no links with other cognitive domains such as inhibitory control. This may be due to differences in the types of games played. For example, action video games have been shown to confer greater cognitive benefits compared to turn‐based strategy or life‐simulation games, which have lower attentional and executive demands (Dale et al. 2020). We did not assess different types of games, which may have obscured associations—a direction for future work. Overall, it is important to note that screen usage is a relatively new construct, and there is still debate over the best way to measure it accurately (Orben 2022). Future work is needed to consider nuances such as types of screen usage, types of media, and individual differences.

Contrary to our hypothesis, the duration of physical activities was not associated with improvements in inhibitory control and attention, information processing speed, or visuospatial sequencing and memory, and was negatively linked to the development of reading and language ability and verbal intellect over time. This is surprising, given that previous literature has consistently demonstrated positive associations between physical activity and cognitive functioning (Erickson et al. 2019; Hapala 2022; Haverkamp et al. 2020; Sibley and Etnier 2003). Differences in measurement may explain the discrepancy. Firstly, earlier studies often examined controlled activities (such as walking, running, stationary cycling, and training in classrooms), which do not fully represent the diverse range of physical activities (such as team sports) that adolescents typically engage in; in addition, types of sports may be associated with development of different cognitive abilities; for example, participation in sports that require mental rotation skills, like wrestling, may benefit cognitive outcomes more compared to running (Moreau et al. 2012). Secondly, earlier studies considered frequency and intensity, whereas we grouped diverse activities and measured only duration, which may have influenced findings. It is also possible that adolescents with higher initial physical activity duration already had higher baseline cognitive scores, leading to smaller observed improvements. Consistent with this interpretation, participants in the top 30% for activity duration had higher baseline cognitive scores than those in the bottom 30% (see Supporting Information: Figure S3). Future research should consider type, intensity, training strategies, and duration when examining associations between physical activities and cognitive outcomes during adolescence.

As hypothesized, longer duration of nonphysical activities was associated with greater improvements in inhibitory control and attention, verbal intellect, and reading and language ability over time. This extends earlier cross‐sectional findings showing links between extracurricular participation and improvements in executive function (Andersen et al. 2019; Diamond 2012), language ability (Ludke 2018), and literacy skills (Hallam 2010). Our findings are also consistent with evidence from intervention studies, which highlight the potential of structured programs to enhance cognitive development. For example, school‐based interventions that promote music, drama, dance, and visual arts (Andersen et al. 2019) have been shown to improve executive functioning and behavioral regulation. However, given that benefits appear to differ by activity type (See and Kokotsaki 2015), future research should explore the differential association of different types of nonphysical activities with cognitive abilities.

We also found sex differences in how the duration of screen use and physical activities are associated with cognitive development. Specifically, the negative association of passive watching with inhibitory control and attention development was more pronounced in females than in males. While previous research suggests that boys spend more time watching TV (Ozmert et al. 2002), it is possible that girls engage with content differently, leading to greater vulnerability. Sex also moderated the association between physical activity and visuospatial sequencing and memory. This could be due to differences in developmental timing and sensitivity to environmental exposures during adolescence (Blakemore et al. 2010; Blakemore and Choudhury 2006; Colom and Lynn 2004; Graber and Petersen 1991). Future studies should investigate these associations during childhood and mid to late adolescence.

While this study provides important insights into the association between modifiable lifestyle factors and cognitive functioning development over time, interpretations must be considered in light of some limitations. First, though this study used a large and diverse sample, it was limited to adolescents from the United States, and the findings may not generalize to other cultural contexts where educational systems differ in how they focus on the development of cognitive skills (Park et al. 1999). Second, some data collected remotely during the COVID‐19 pandemic may have been influenced by the circumstances of data collection, despite accounting for the type of visit (Saragosa‐Harris et al. 2022). Moreover, differential attrition in the ABCD sample (Feldstein Ewing et al. 2022; Rakesh et al. 2025b) could bias the findings towards higher SES adolescents; additionally, missingness on the physical activities variable could impact the reliability of our results. Finally, only five of the original seven baseline tests were repeated at follow‐up, limiting our ability to assess the full range of cognitive abilities. Furthermore, we did not investigate any mechanisms behind the associations. Future studies should explore the underlying neurobiological mechanisms driving these associations. Additionally, examining other environmental and lifestyle factors, such as diet and social interactions, could provide a more comprehensive understanding of how to support optimal cognitive development during this critical developmental period. Finally, although the longitudinal design allows us to examine developmental trajectories over time, causal inferences cannot be made with an observational study design, and residual confounding is likely. Future studies should incorporate experimental designs and causal frameworks.

In conclusion, this study highlights the association between modifiable lifestyle factors (i.e., sleep duration, screen time, extracurricular activities) and changes in cognitive abilities during adolescence. The findings underscore the importance of promoting healthy lifestyle habits early in adolescence, as these factors may have long‐term implications for cognitive development.

## Supporting information

Supporting Materials revise2.
