# Supplementary material for: Associations of Sleep, Screen Time, and Extracurricular Activities With Cognitive Development: A Longitudinal Study
Source: J Adolesc. 2025 Nov 4;98(2):394–406. doi: 10.1002/jad.70069 (PMC12894487; doi:10.1002/jad.70069)

**Supplementary Material**

Table of Contents

[Figure S1: Histogram of developmental factors, cognitive functioning, and covariates (income-to-needs ratio and average parent education) 2](#_Toc212901852)

[Activities included in the physical and non-physical categories and calculation 4](#_Toc212901853)

[Figure S2: Correlation plot, relationship between modifiable lifestyle factors, cognitive functioning, and covariates (income-to-needs ratio and average parent education) 5](#_Toc212901854)

[Table S1: Model output for normative change in cognitive function 6](#_Toc212901855)

[Table S2: Model fit statistics for the model output for modifiable lifestyle factors and FCF development 7](#_Toc212901856)

[Table S3: Model fit statistics for the model output for modifiable lifestyle factors and FCF development 9](#_Toc212901857)

[Table S4: Moderating role of sex in the association between lifestyle factors and FCF development 10](#_Toc212901858)

[Table S5: Moderating role of sex in the association between lifestyle factors and CCF development 12](#_Toc212901859)

[Table S6: Model fit statistics for modifiable lifestyle factors and cognitive functioning development by sex 14](#_Toc212901860)

[Figure S3: Baseline Oral Reading and Picture Vocabulary Scores by Physical Activity Group (Top vs Bottom 30%) 15](#_Toc212901861)

### Figure S1: Histogram of developmental factors, cognitive functioning, and covariates (income-to-needs ratio and average parent education)

Modifiable lifestyle factors

Cognitive functioning tasks baseline year

Cognitive function tasks 2-year follow up

Covariates

### Activities included in the physical and non-physical categories and calculation

- Physical Activities: ballet/dance, baseball/softball, basketball, climbing, field hockey, football, gymnastics, ice hockey, horseback riding/polo, ice or inline skating, martial arts, lacrosse, rugby, skateboarding, skiing/snowboarding, soccer, surfing, swimming/water polo, tennis, track/running/cross-country, wrestling/mixed martial arts, volleyball, yoga/Tai Chi.
- Non-physical Activities: musical instruments (singing/choir/guitar/piano/drums/violin/flute/band/rock band/orchestra), drawing/painting/graphic art/photography/pottery/sculpting, drama/theatre/acting/film, crafts like knitting/building model cars or airplanes, competitive games like chess/cards/darts, and hobbies like collecting stamps or coins.

Calculation method:

Weekly activity time was calculated based on the product of the number of sessions per week and the duration of each session. The total weekly activity time per category (i.e., physical vs non-physical) was then determined by summing the calculated times for all activities in each category.

### Figure S2: Correlation plot, relationship between modifiable lifestyle factors, cognitive functioning, and covariates (income-to-needs ratio and average parent education)


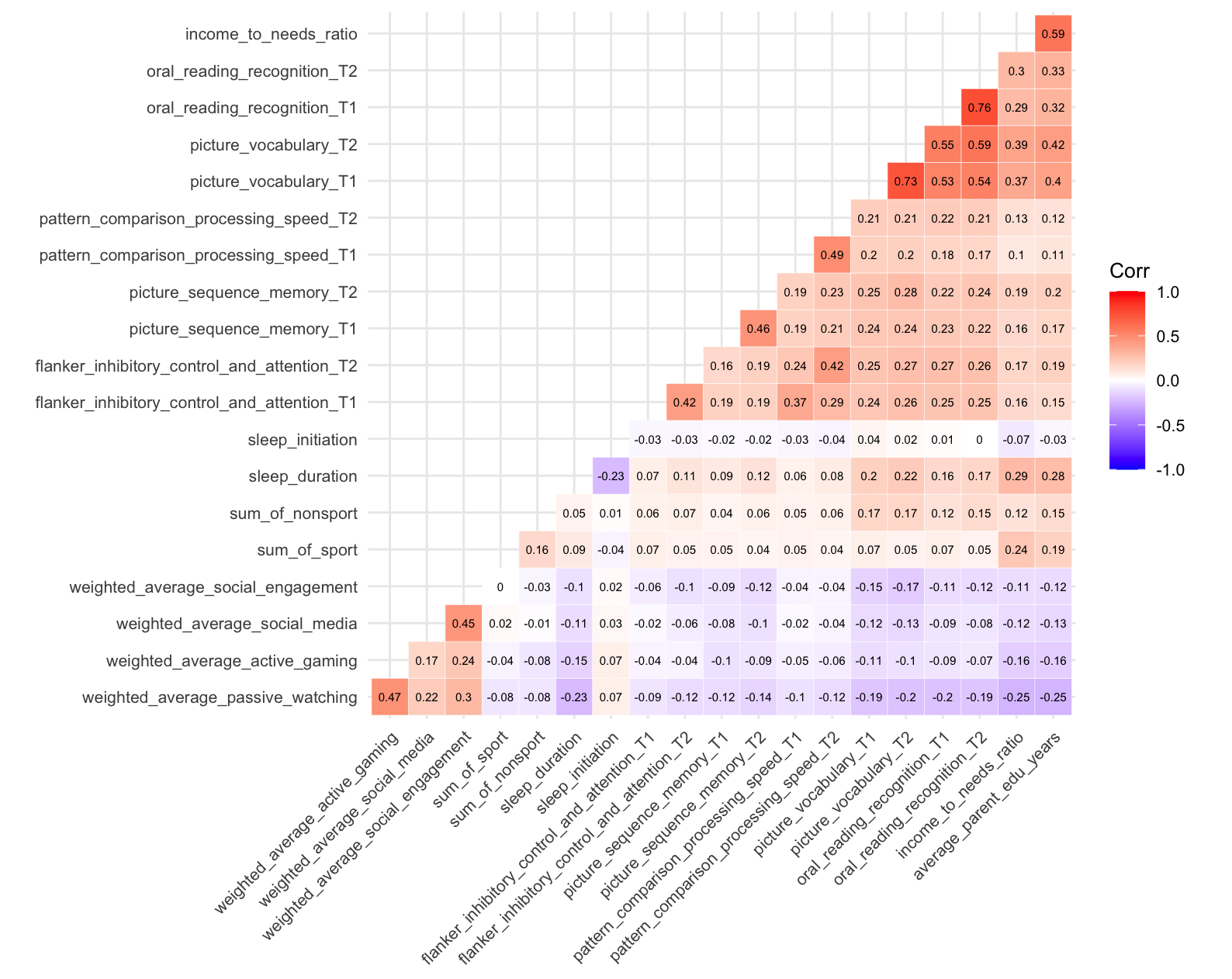


### Table S1: Model output for normative change in cognitive function

| **Cognitive function** | **Coefficient** | **Standard error** | **p value** |
| --- | --- | --- | --- |
| Inhibitory control and attention | 5.99 | 0.10 | <0.001 |
| Information processing speed | 15.34 | 0.16 | <0.001 |
| Visuospatial sequencing and memory | 5.60 | 0.13 | <0.001 |
| Verbal intellect | 4.30 | 0.06 | <0.001 |
| Reading and language ability | 3.95 | 0.05 | <0.001 |

### Table S2: Model fit statistics for the model output for modifiable lifestyle factors and FCF development

| **Predictor** | **Outcome** | **Partial R²** | **Conditional R²** | **AIC** | **BIC** |
| --- | --- | --- | --- | --- | --- |
| Passive watching | Inhibitory control and attention | 0.000 | 0.34 | 48873 | 48956 |
| Gaming |  | 0.000 | 0.34 | 48870 | 48953 |
| Social media |  | 0.000 | 0.34 | 48919 | 49002 |
| Social engagement |  | 0.000 | 0.34 | 48806 | 48889 |
| Physical |  | 0.000 | 0.34 | 49057 | 49140 |
| Non-physical |  | 0.001 | 0.34 | 49050 | 49133 |
| Sleep duration |  | 0.000 | 0.34 | 49048 | 49130 |
| Sleep initiation |  | 0.000 | 0.34 | 49069 | 49152 |
| Passive watching | Information processing speed | 0.002 | 0.35 | 58304 | 58387 |
| Gaming |  | 0.000 | 0.35 | 58300 | 58383 |
| Social media |  | 0.000 | 0.35 | 58356 | 58439 |
| Social engagement |  | 0.000 | 0.35 | 58253 | 58336 |
| Physical |  | 0.000 | 0.34 | 58475 | 58557 |
| Non-physical |  | 0.000 | 0.34 | 58472 | 58555 |
| Sleep duration |  | 0.001 | 0.34 | 58470 | 58553 |
| Sleep initiation |  | 0.000 | 0.34 | 58481 | 58564 |
| Passive watching | Visuospatial sequencing and memory | 0.000 | 0.32 | 71259 | 71344 |
| Gaming |  | 0.000 | 0.32 | 71253 | 71338 |
| Social media |  | 0.001 | 0.32 | 71288 | 71374 |
| Social engagement |  | 0.002 | 0.32 | 71165 | 71251 |
| Physical |  | 0.000 | 0.31 | 71420 | 71506 |
| Non-physical |  | 0.000 | 0.31 | 71418 | 71504 |
| Sleep duration |  | 0.001 | 0.31 | 71408 | 71494 |
| Sleep initiation |  | 0.000 | 0.31 | 71428 | 71514 |

### Table S3: Model fit statistics for the model output for modifiable lifestyle factors and FCF development

| **Predictor** | **Outcome** | **Partial R²** | **Conditional R²** | **AIC** | **BIC** |
| --- | --- | --- | --- | --- | --- |
| Passive watching | Verbal intellect | 0.000 | 0.62 | 58289 | 58375 |
| Gaming |  | 0.000 | 0.62 | 58303 | 58388 |
| Social media |  | 0.000 | 0.62 | 58322 | 58408 |
| Social engagement |  | 0.002 | 0.62 | 58205 | 58291 |
| Physical |  | 0.002 | 0.62 | 58404 | 58490 |
| Non-physical |  | 0.001 | 0.62 | 58418 | 58504 |
| Sleep duration |  | 0.002 | 0.62 | 58407 | 58493 |
| Sleep initiation |  | 0.000 | 0.62 | 58443 | 58529 |
| Passive watching | Reading and language ability | 0.000 | 0.64 | 52964 | 53050 |
| Gaming |  | 0.000 | 0.64 | 52963 | 53049 |
| Social media |  | 0.000 | 0.64 | 52996 | 53081 |
| Social engagement |  | 0.000 | 0.64 | 52914 | 52999 |
| Physical |  | 0.001 | 0.64 | 53064 | 53150 |
| Non-physical |  | 0.001 | 0.64 | 53056 | 53142 |
| Sleep duration |  | 0.001 | 0.65 | 53069 | 53154 |
| Sleep initiation |  | 0.000 | 0.64 | 53088 | 53174 |

### Table S4: Moderating role of sex in the association between lifestyle factors and FCF development

| **Predictor** | **Outcome** | **b value** | **SE value** | **t value** | **p value** | **pFDR** | **Partial R²** | **Conditional R²** | **AIC** | **BIC** |
| --- | --- | --- | --- | --- | --- | --- | --- | --- | --- | --- |
| Passive watching | Inhibitory control and attention | -0.28 | 0.09 | -3.03 | 0.002 | 0.020* | 0.000 | 0.34 | 48869 | 48959 |
| Gaming |  | -0.05 | 0.16 | -0.32 | 0.753 | 0.803 | 0.000 | 0.34 | 48874 | 48964 |
| Social media |  | -0.10 | 0.41 | -0.25 | 0.803 | 0.803 | 0.000 | 0.34 | 48921 | 49011 |
| Social engagement |  | -0.21 | 0.20 | -1.04 | 0.298 | 0.559 | 0.000 | 0.34 | 48808 | 48898 |
| Physical |  | 0.04 | 0.02 | 1.68 | 0.092 | 0.369 | 0.000 | 0.34 | 49062 | 49152 |
| Non-physical |  | 0.02 | 0.04 | 0.38 | 0.705 | 0.803 | 0.000 | 0.34 | 49056 | 49146 |
| Sleep duration |  | 0.19 | 0.15 | 1.25 | 0.210 | 0.559 | 0.000 | 0.34 | 49050 | 49140 |
| Sleep initiation |  | -0.01 | 0.01 | -0.94 | 0.349 | 0.559 | 0.000 | 0.34 | 49077 | 49166 |
| Passive watching | Information processing speed | -0.07 | 0.18 | -0.38 | 0.702 | 0.916 | 0.000 | 0.35 | 58308 | 58398 |
| Gaming |  | -0.06 | 0.31 | -0.20 | 0.842 | 0.916 | 0.000 | 0.35 | 58302 | 58392 |
| Social media |  | -0.33 | 0.80 | -0.41 | 0.683 | 0.916 | 0.000 | 0.35 | 58357 | 58446 |
| Social engagement |  | 0.43 | 0.39 | 1.12 | 0.262 | 0.916 | 0.000 | 0.35 | 58254 | 58344 |
| Physical |  | 0.11 | 0.04 | 2.37 | 0.018 | 0.141 | 0.000 | 0.34 | 58475 | 58565 |
| Non-physical |  | 0.03 | 0.09 | 0.39 | 0.695 | 0.916 | 0.000 | 0.34 | 58477 | 58567 |
| Sleep duration |  | -0.03 | 0.29 | -0.11 | 0.916 | 0.916 | 0.000 | 0.34 | 58473 | 58562 |
| Sleep initiation |  | 0.01 | 0.03 | 0.46 | 0.644 | 0.916 | 0.000 | 0.34 | 58488 | 58578 |
| Passive watching | Visuospatial sequencing and memory | -0.24 | 0.13 | -1.80 | 0.071 | 0.285 | 0.000 | 0.32 | 71260 | 71353 |
| Gaming |  | -0.29 | 0.24 | -1.20 | 0.230 | 0.457 | 0.000 | 0.32 | 71254 | 71347 |
| Social media |  | -0.05 | 0.59 | -0.09 | 0.927 | 0.927 | 0.000 | 0.32 | 71290 | 71382 |
| Social engagement |  | 0.30 | 0.28 | 1.07 | 0.286 | 0.457 | 0.000 | 0.32 | 71167 | 71260 |
| Physical |  | 0.10 | 0.03 | 2.99 | 0.003 | 0.022* | 0.000 | 0.32 | 71418 | 71511 |
| Non-physical |  | -0.09 | 0.06 | -1.38 | 0.167 | 0.445 | 0.000 | 0.31 | 71422 | 71515 |
| Sleep duration |  | 0.20 | 0.22 | 0.93 | 0.354 | 0.473 | 0.000 | 0.31 | 71411 | 71504 |
| Sleep initiation |  | -0.02 | 0.02 | -0.78 | 0.438 | 0.500 | 0.000 | 0.31 | 71435 | 71528 |

### Table S5: Moderating role of sex in the association between lifestyle factors and CCF development

| **Predictor** | **Outcome** | **b value** | **SE value** | **t value** | **p value** | **pFDR** | **Partial R²** | **Conditional R²** | **AIC** | **BIC** |
| --- | --- | --- | --- | --- | --- | --- | --- | --- | --- | --- |
| Passive watching | Verbal intellect | -0.09 | 0.07 | -1.34 | 0.181 | 0.241 | 0.000 | 0.62 | 58293 | 58386 |
| Gaming |  | 0.25 | 0.12 | 2.07 | 0.039 | 0.203 | 0.000 | 0.62 | 58303 | 58396 |
| Social media |  | 0.52 | 0.30 | 1.74 | 0.082 | 0.218 | 0.000 | 0.62 | 58322 | 58415 |
| Social engagement |  | 0.22 | 0.14 | 1.59 | 0.112 | 0.224 | 0.000 | 0.62 | 58207 | 58300 |
| Physical |  | 0.02 | 0.02 | 1.41 | 0.160 | 0.241 | 0.000 | 0.62 | 58410 | 58503 |
| Non-physical |  | -0.03 | 0.03 | -0.98 | 0.325 | 0.325 | 0.000 | 0.62 | 58424 | 58517 |
| Sleep duration |  | 0.22 | 0.11 | 1.95 | 0.051 | 0.203 | 0.000 | 0.62 | 58408 | 58501 |
| Sleep initiation |  | 0.01 | 0.01 | 1.01 | 0.315 | 0.325 | 0.000 | 0.62 | 58452 | 58545 |
| Passive watching | Reading and language ability | -0.07 | 0.05 | -1.35 | 0.177 | 0.430 | 0.000 | 0.64 | 52969 | 53061 |
| Gaming |  | 0.03 | 0.09 | 0.32 | 0.751 | 0.751 | 0.000 | 0.64 | 52968 | 53061 |
| Social media |  | -0.12 | 0.23 | -0.55 | 0.584 | 0.700 | 0.000 | 0.64 | 52999 | 53091 |
| Social engagement |  | -0.08 | 0.11 | -0.74 | 0.461 | 0.700 | 0.000 | 0.64 | 52918 | 53010 |
| Physical |  | 0.02 | 0.01 | 1.34 | 0.180 | 0.430 | 0.000 | 0.64 | 53071 | 53164 |
| Non-physical |  | -0.01 | 0.02 | -0.51 | 0.613 | 0.700 | 0.000 | 0.64 | 53064 | 53156 |
| Sleep duration |  | 0.10 | 0.08 | 1.24 | 0.215 | 0.430 | 0.000 | 0.65 | 53072 | 53165 |
| Sleep initiation |  | -0.01 | 0.01 | -1.31 | 0.190 | 0.430 | 0.000 | 0.64 | 53096 | 53189 |

### Table S6: Model fit statistics for modifiable lifestyle factors and cognitive functioning development by sex

| **Predictor** | **Outcome** | **Sex** | **Partial R²** | **Conditional R²** | **AIC** | **BIC** |
| --- | --- | --- | --- | --- | --- | --- |
| Passive watching | Inhibitory control and attention | Male | 0.000 | 0.43 | 25896 | 25959 |
|  |  | Female | 0.012 | 0.28 | 23027 | 23088 |
| Physical extracurricular activities | Visuospatial sequencing and memory | Male | 0.000 | 0.33 | 37331 | 37396 |
|  |  | Female | 0.012 | 0.33 | 34157 | 34221 |

### Figure S3: Baseline Oral Reading and Picture Vocabulary Scores by Physical Activity Group (Top vs Bottom 30%)

Baseline Picture Vocabulary Scores by Physical Activity Group


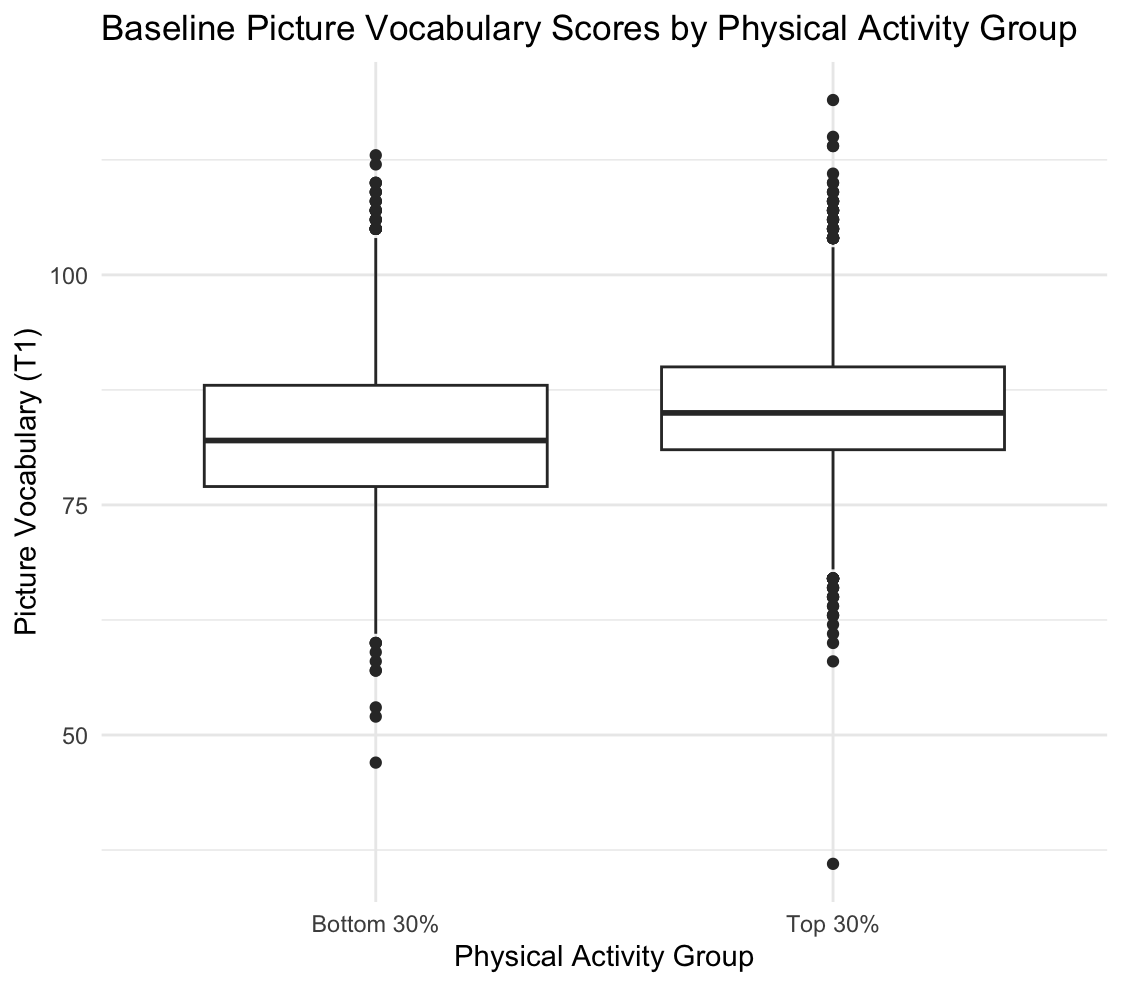


Baseline Oral Reading Scores by Physical Activity Group
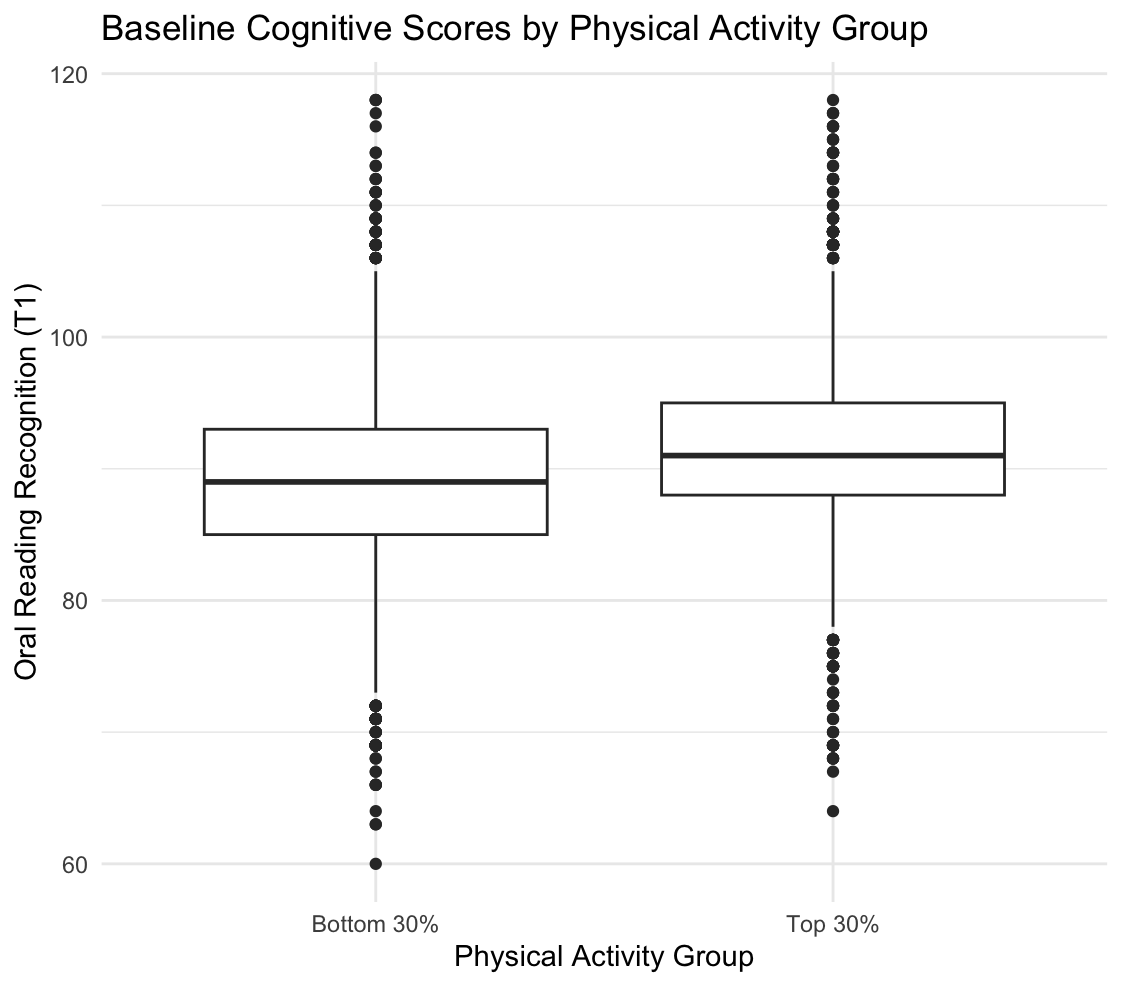

Supplement: Supplementary file 1 — Supporting Materials revise2. [file JAD-98-394-s001.docx]
